# Supplementary material for: The causal relationship between the human gut microbiota and pyogenic arthritis: a Mendelian randomization study
Source: Front Cell Infect Microbiol. 2024 Nov 26;14:1452480. doi: 10.3389/fcimb.2024.1452480 (PMC11629706; doi:10.3389/fcimb.2024.1452480)
Supplement: Supplementary file 1 [file DataSheet1.zip › mendelian test/ebi-a-GCST90027560.csv_forest.pdf]

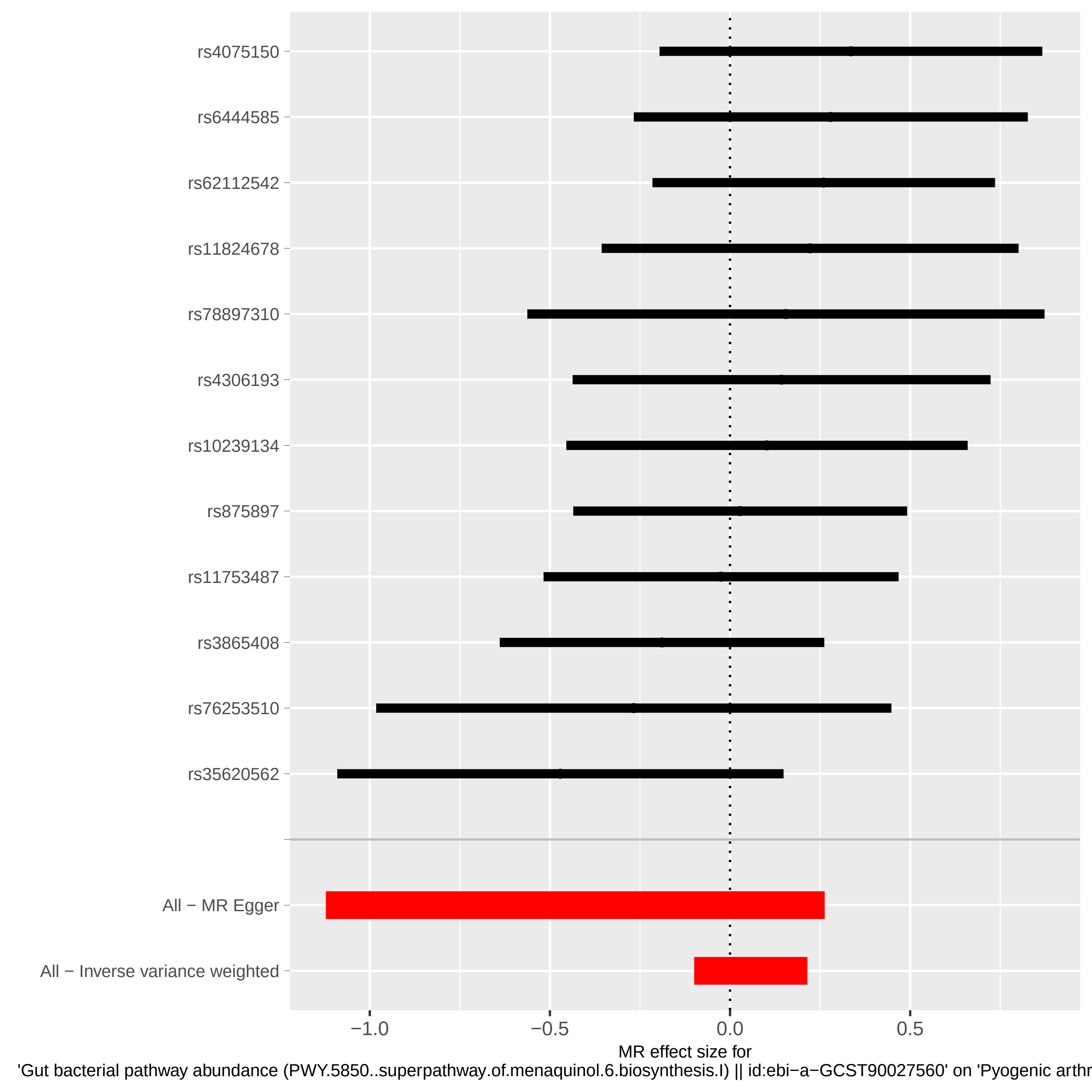

rs4075150
rs6444585
rs62112542
rs11824678
rs78897310
rs4306193
rs10239134
rs875897
rs11753487
rs3865408
rs76253510
rs35620562
All − MR Egger
All − Inverse variance weighted
−1.0
−0.5
0.0
0.5
MR effect size for
'Gut bacterial pathway abundance (PWY.5850..superpathway.of.menaquinol.6.biosynthesis.I) || id:ebi−a−GCST90027560' on 'Pyogenic arthr
